# Incidence, predisposing factors, management and survival following cardiac arrest due to subarachnoid haemorrhage: a review of the literature

**DOI:** 10.1186/1757-7241-20-75

**Published:** 2012-11-14

**Authors:** Markus B Skrifvars, Michael J Parr

**Affiliations:** 1 Department of Anaesthesiology and Intensive Care Medicine, Helsinki University Hospital, Helsinki, Finland; 2 Intensive Care Unit, Liverpool Hospital, Sydney, Australia; 3 University of New South Wales, Sydney, Australia

## Abstract

**Introduction:**

The prevalence of cardiac arrest among patients with subarachnoid haemorrhage [SAH], and the prevalence of SAH as the cause following out-of-hospital cardiac arrest [OHCA] or in-hospital cardiac arrest [IHCA] is unknown. In addition it is unclear whether cardiopulmonary resuscitation [CPR] and post-resuscitation care management differs, and to what extent this will lead to meaningful survival following cardiac arrest [CA] due to SAH.

**Aim:**

We reviewed the literature in order to describe; 1.The prevalence and predisposing factors of CA among patients with SAH 2.The prevalence of SAH as the cause of OHCA or IHCA and factors characterising CPR 3.The survival and management of SAH patients with CA.

**Material and methods:**

The following sources, PubMed, CinAHL and The Cochrane DataBase were searched using the following Medical Subheadings [MeSH]; 1. OHCA, IHCA, heart arrest and 2. subarachnoid haemorrhage. Articles containing relevant data based on the abstract were reviewed in order to find results relevant to the proposed research questions. Manuscripts in other languages than English, animal studies, reviews and case reports were excluded.

**Results:**

A total of 119 publications were screened for relevance and 13 papers were included. The prevalence of cardiac or respiratory arrest among all patients with SAH is between 3-11%, these patients commonly have a severe SAH with coma, large bleeds and evidence of raised intracerebral pressure on computed tomography scans compared to those who did not experience a CA. The prevalence of patients with SAH as the cause of the arrest among OHCA cases vary between 4 to 8% among those who die before hospital admission, and between 4 to 18% among those who are admitted. The prevalence of SAH as the cause following IHCA is low, around 0.5% according to one recent study. In patients with OHCA survival to hospital discharge is poor with 0 to 2% surviving. The initial rhythm is commonly asystole or pulseless electrical tachycardia. In IHCA the survival rate is variable with 0-27% surviving. All survivors experience brief cardiac arrests with short latencies to ROSC.

**Conclusion:**

Cardiac arrest is a fairly common complication following severe SAH and these patients are encountered both in the pre-hospital and in-hospital setting. Survival is possible if the arrest occurs in the hospital and the latency to ROSC is short. In OHCA the outcome seems to be uniformly poor despite initially successful resuscitation.

## Introduction

Subarachnoid haemorrhage (SAH) is a devastating disease that may lead to sudden death [1]. The reported proportion of SAH patients that die prior to seeking medical attention is in the range of 3-21%, and sudden death seems to be more common in SAH due to aneurysms of the posterior circulation [2]. Proposed mechanisms for cardiac arrest in SAH patients include cardiac arrhythmias, respiratory arrest and cerebral herniation due to raised intracranial pressure [2].

The role of resuscitation in these patients is unclear and survival, especially long term survival in patients with SAH and cardiac arrest varies considerably in published studies [3,4]. Differences are likely to be related to a number of issues such as; different study settings with some including only out-of-hospital cardiac arrests (OHCA) and some in-hospital cardiac arrest (IHCA) and study sample with some studies including all attempted resuscitations and some including only OHCA patients surviving to hospital admission. The present study attempted to systematically review and describe characteristics, survival and factors associated with survival in patients with SAH experiencing a cardiac arrest requiring cardiopulmonary resuscitation.

## Materials and methods

We defined the following research questions:

1. What is the prevalence of CA among patients with SAH and are there predisposing factors?

2. What is the prevalence of SAH as the cause of the arrest among patients with OHCA or IHCA who are resuscitated?

3. How are SAH patients with CA managed and what is the survival?

**Figure 1:**
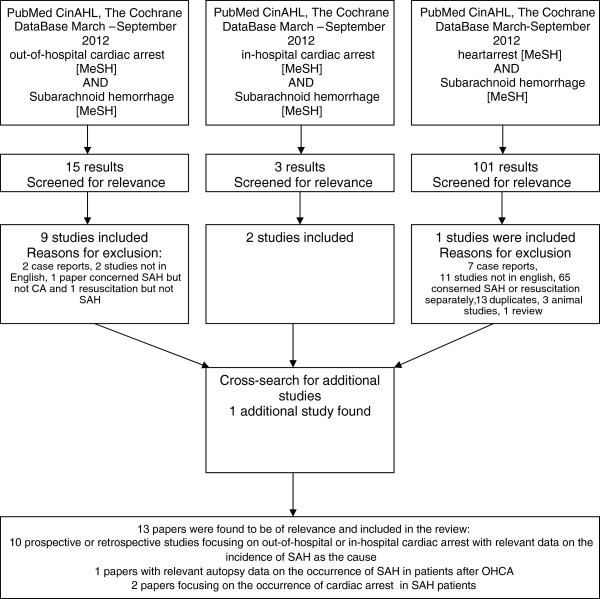
Applied search strategy and results.

In order to answer these proposed questions we undertook a search of the literature using the following Medical Subheadings (MeSH); out-of-hospital cardiac arrest, in-hospital cardiac arrest, heart arrest OR AND subarachnoid haemorrhage (Figure 1). Manuscripts in other languages than English, animal studies and case reports were excluded. The following search engines were used PubMED, CiNAHL, the Cochrane Database. In addition, all included papers were searched for valid cross-references. Recent numbers (September 2012) of scientific journals were also checked for suitable publications.

The titles and abstracts were screened for relevance, and full texts were obtained if found relevant. Included papers were reviewed in order to find data regarding the proposed research questions. The literature search was performed by one of the authors (MBS) after which results and conclusions were independently checked by the other author (MP). In some papers proportion of patients with SAH were calculated based on provided results. We made no attempt to contact the authors of the included papers in order to confirm or to obtain additional data. A differentiation was made between “assumed” and “confirmed” SAH since it was considered likely to variability in reporting cause based on clinical details or those verified with autopsy or radiological studies.

## Results

The initial searches yielded a total of 119 publications (Figure 1). These papers were screened for relevance, and cross references were searched for, after which 13 papers were included in the final analysis. Most included papers were from single institutions, apart from registry data from Australia and Canada [5,6]. The geographical distribution of published papers included several papers Japan and one paper from Australia, North-America and Europe respectively.

### The prevalence of cardiac arrest among SAH patients

A Canadian Stroke Registry recently reported that among a registry of SAH patients treated a 12 different hospital in Canada between 2003 and 2008, 7% experienced cardiac or respiratory arrest (Table 1) [6]. Slightly lower figures of 4% were reported from a single centre study from US including 576 SAH Patients, but included only patients with a successful resuscitation [7]. Inamasu reported that among 315 patients admitted to a tertiary centre in Japan as much as 11% had experienced cardiac arrest (Table 1) [8]. Toussaint and colleagues on the other hand reported a case series of 305 patients, of which 3.6% suffered a cardiac arrest either outside or inside the hospital [3]. 

**Table 1:** The prevalance of cardiac arrest and contributing factors in studies including series of patients with subarachnoid haemorrhage

**Study**	**Years**	**Setting**	**Included patients**	**Proportion of SAH patients that had a CA**	**Factors associated with the occurrence of CA**	**Survival of SAH patients with CA**	**Factors associated with survival in those with CA**	
Wartenberg et al.	1996-2002	Tertiary hospital in the US	CT or cerebrospinal fluid analysis	21 (4%) of 546 SAH patients	NR	NR	NR	
Toussaint LG et al.	1990-1997	Tertiary hospital	All admitted SAH patients	11 (3.6%) out of 305 SAH patients (5 IHCA, 5 OHCA, 1 EMS witnessed)	Low GCS, high Hunt Hess grade. aneurysm rupture	6 (55%) out of 11 survived, 3 (27%) with good outcome	All survivors had a brief cardiac arrests requiring only defibrillation	
Inamasu et al.	2003-2009	Japanese tertiary centre	Admitted SAH patients	11% of 315 SAH patients	NR	0% survived	NR	
Vergouwen et al.	2003 to 2008	Registry of the Canadian Stroke Network	SAH patients treated at 11 hospitals	64/931 (6.6%) had a cardiac or respiratory arrest	NR	CA patients had lower survival than those without (odds ratio 7.2 (2.0-25.7)	NR	

### The prevalence of SAH as the cause in resuscitated OHCA and IHCA patients

No study reported the prevalence of SAH as the cause of all attempted resuscitations of OHCA patients, i.e. including those terminated in the field. Two studies reported the proportion of SAH as the cause among resuscitated patients that survived to hospital admission: Kürkciyan and colleagues reported that from a total of 765 OHCA patients admitted to the hospital in Vienna in Austria, 4% were found to have SAH as the cause (Table 2) [9]. The diagnosis was made using computed tomography (CT) scan of the brain in 20 patients and following autopsy in 3 patients. Mitsuma and colleagues reported 6% prevalence of SAH among 244 OHCA patients admitted to a Japanese tertiary hospital [4]. All cases were diagnosed using CT scan. Inamasu and collegues also reported data from a tertiary centre in Japan and found that 16% out of 142 patients admitted following witnessed OHCA had SAH as the cause of the arrest [10]. Cochi, on the other hand found only one case (2%) of SAH among 51 OHCA patients in whom a CT brain was performed after hospital admission [11]. 

**Table 2:** Studies reporting outcome of out-of-hospital or in-hospital cardiac arrest in which the prevalence of subarachnoid haemoorhage as the cause of the arrest was reported

**Study**	**Years**	**Setting**	**Included patients**	**Method for SAH diagnosis**	**Proportion OHCA/IHCA with SAH**	**Typical features of SAH patients**	**Resuscitation factors associated with SAH**	**Proportion of patients with achieved ROSC**	**Survival in patients with SAH and CA**	**Comment**
Shapiro S	1989-96	Trauma centre in the US	Patients with respiratory or cardiac arrest and documented SAH	CT	NR		NR	NR	43% survival, 20% with good neurologic outcome	Does not report separate survival for respiratory/cardiac arrest
Kürkciyan et al.	1991-99	Mid-sized urban city, OHCA registry	OHCA patients with ROSC admitted to the ED	CT and autopsy	27 (4%) out of 765 (all OHCA aetiologies)	Age<40, headache, female gender	PEA/ASY in 93%	NR	1 (4%) survived to hospital discharge	
Inamasu et al.	2004-7	Japanese tertiary centre	OHCA patients admitted to the ED with CT brain performed	CT	26 (18%) out of 142 OHCA patients with CT brain had SAH	Female gender, headache	PEA/ASY in 96%	NR	0 (0%) survived to hospital discharge	All patients had ROSC
Mitsuma et al.	2007-9	Japanese tertiary centre	OHCA patients transported to the ED	CT (including post-mortem CT)	14 (6%) of 244 OHCA patients	Female gender	PEA/ASY in 100%	10(71%) out of 14	0 (0%) survived to hospital discharge	
Cocchi et al.	2006-9	US tertiary care centre	OHCA admitted to the ED who had CT brain performed	CT	1 (2%) out of 51 patients (CT Brain done) had confirmed SAH	NR	NR	NR	NR	
Inamasu et al.	2007	Japanese tertiary centre	OHCA patients admitted to a tertiary centre		10 (8%) out of 124 OHCA patients had SAH on a CT brain		Female gender	10 (100%) out of 10 patients had ROSC	0 (0%) survived to hospital discharge	
Yuzawa et al.	2003-7	Japanese tertiary centre	CA patients with CT brain	CT and autopsy	8 (18%) out of 45 patients had SAH	NR	NR	NR	NR	Focus on the paper is radiological features of post CA CT findings
Wallmuller et al.	1991-08	ED ICU	Patients resuscitated from IHCA treated in the ED ICU	CT	5 (0.5%) out of 1041 patients had SAH	NR	NR	NA	0%	Only patients with ROSC included

OHCA=out-of-hospital cardiac arrest, IHCA-in-hospital cardiac arrest, CT=computed tomography, NR=not reported, ED=emergency department, NA=not applicable, CA=cardiac arrest.

One recent paper by Walmuller and colleagues reported data from their IHCA registry [12]. The registry consists of data from successfully resuscitated IHCA patients who had their arrest in the emergency department, in the radiology department, in a public place within the hospital, or admitted after having had an IHCA in another hospital (Table 2) [12]. Out of 1041 patients 5 (0.5%) had SAH as the cause [12].

### The prevalence of SAH in OHCA patients with unsuccessful resuscitation who underwent autopsy

The proportion of patients with SAH as the cause following unsuccessful resuscitation in the pre-hospital setting was reported to be 8% according Australian registry data [5]. Noteworthy is that this paper by Deasy and colleagues included only patients aged between 16 and 39 years.

### Patient characteristics, pre-arrest symptoms and factors at resuscitation of patients with SAH and CA

Female gender seemed to be more common than male among OHCA patients and SAH, with proportions ranging from 63% to 78% [4,9]. Headache seemed to be a common preceding sign with 39% to 48% of SAH patients complaining of headache prior to the arrest [9,11,13].

The initial rhythm was most commonly pulseless electrical activity (PEA) with proportions ranging from 43% to 63% [4,8,9] and asystole, 44% to 57% [4,8,9] in OHCA. Ventricular fibrillation [VF] occurred in 0 to 18% [3,4,8,9].

Two studies reported the proportion of patients achieving ROSC following OHCA and SAH; Mitsuma reported that ROSC was achieved in 10 (71%) out of 14 OHCA patients with SAH [4]. Return of spontaneous circulation was more commonly achieved than with other types of intracranial bleed and in those with a cardiac cause [4]. Inamasu reported that among 23 patients with SAH and OHCA, ROSC was achieved in all 23 cases, in three patients prior to hospital admission and in 20 in the hospital [10]. In one study by Mitsuma and colleagues ROSC rates were higher in SAH patients than with other aetiologies [4].

Latency to ROSC following OHCA in SAH patients was reported in 2 studies and ranging from 22 to 26 minutes [14,15]. In the study by Toussiant and collegues of SAH patients with either OHCA or IHCA latencies to ROSC ranged from 1 to 20 minutes [3].

### Outcome and factors contributing to outcome following resuscitation of CA patients with SAH

Five studies reported survival to hospital discharge rates of OHCA patients with SAH [3,4,8-10]. Outcome was very poor, of a total of 82 OHCA patients [calculated from the individual studies] 4 survived [6%] of whom at least 2 were reported to be in a poor neurological state (Tables 1 and 2). All survivors had a brief cardiac arrest and seemed to require only defbrillation [3]. All patients who required prolonged CPR [>10 minutes] had a poor outcome.

We identified a total of 10 IHCA patients with SAH that were resuscitated [3,12]. Of these 2 survived, one with good neurological recovery [3]. The survivor experienced a brief cardiac arrest (1 minute) [3]

Shapiro and colleagues reported 26 cases of patients with SAH with either respiratory or cardiac arrest outside of the hospital [16]. Of these cases, only six patients received chest compressions indicating complete cardiac arrest. The outcome in this sample was remarkably good with a survival rate of 20%. The outcome of those requiring chest compressions is not reported in the study [13].

### Diagnosis of SAH in patients following cardiac arrest

Computed tomography (CT] scan seems to the most commonly used method of diagnosing SAH in patients with SAH [4,9,10]. Interestingly Yuzawa and colleagues showed in patients with severe brain oedema following hypoxic brain injury that the CT scan may show a “pseudo SAH” appearance [17]. This occurred in 20% of patients of a sample of patients with cardiac arrest and the absence of SAH was verified on autopsy. This finding occurred within 3 days from the incident and was associated with a poor prognosis. The use of CT angiography and cerebral fluid specimen examination may be used to verify the existence of SAH, but its role during the post-resuscitation phase needs to be elucidated. Inamasu and colleagues measured cardiac troponin in OHCA survivors and compared those with SAH as the cause and those without [10]. They found that SAH patients have lower initial troponin levels than those experiencing an OHCA due to a cardiac cause.

### Type and severity of SAH and the incidence of CA

In SAH patients cardiac arrest seems to occur during the ictus, during rebleeds and in some cases in relation to cardiac complications such as myocardial ischaemia [3]. Most SAH patients experiencing a prolonged cardiac arrest were deeply comatose on arrival to the hospital with a Hunt and Hess grade of 5 [4,8-10]. On the other hand patients experiencing a brief cardiac arrest had GCS of around 8 to 10 [3]. In the study by Shapiro and colleagues patients who experienced a cardiorespiratory arrest had SAH with thick clots and evidence of increased intracerebral pressure [ICP] [16]. Similar findings were presented by Mitsuma and Inamasu, with all CA patients having SAH with Fischer grades of 3 or 4 on CT brain scans [4,10]. In the study by Shapiro there was some indication that aneurysms in the posterior circulation compared to the anterior circulation predisposes to a respiratory or cardiac arrest [8]. This was, however, not the case in study by Toussiant and colleagues [3].

### Treatment of SAH patients with CA

Only Toissiant and colleagues detailed CA management of SAH patients; patients were managed according to standard basic and advanced life support guidelines including adrenaline, atropine and defibrillation [3]. Neurosurgical care was undertaken in 0 to 55% of cases [3,4,8,9]. In some cases care was limited to conservative neurosurgical intensive care without surgery [9]. Between 50 to 100% of patients were in some studies treated with clipping [3,16]. In some other studies measures to treat raised intracranial pressure using ventricular drains were taken, but aneurysm surgery was not undertaken due to perceived poor prognosis [8].

## Discussion

The present review indicates that between 4-7% of SAH experience a CA. Among OHCA patients between 4-7% have an arrest related to SAH probably higher than in IHCA were the only study available reported only 0.5% of IHCAs are due to SAH. Patients with SAH and CA are commonly of female gender and younger than the average CA patients. Even though resuscitation attempts are initially successful and ROSC is achieved, survival is dismal following prolonged resuscitation attempts. Survival is possible in brief cardiac arrests only requiring defibrillation.

It is well known that some patients with SAH die due to sudden death [1]. In a meta-analysis from 2002 the incidence of sudden death in patients with SAH was reported to be between 11-14% [1]. This is higher than the reported rate of CA among hospital cohorts of SAH patients suggesting that a considerable proportion of SAH patients die outside of the hospital. How many of these undergo resuscitation attempts are difficult to determine since none of the studies included in the present review included autopsy data of patients terminated in the field and data of those resuscitated and admitted to the hospital. On the other hand, the practise in most Asian Emergency Medical Service systems seems to be that all patients found in OHCA are transported to the hospital with ongoing CPR [18]. Therefore the prevalence of SAH in OHCA of 6-18% reported by Inamasu and Mitsuma may be representative of the SAH prevalence OHCA patients in Japan [4,8]. These rates are higher than reported in Europe and may reflect the high incidence of SAH in Japan [14].

Cardiac involvement ranging from benign ECG changes to cardiogenic shock and ventricular arrhythmias have been reported following SAH [19,20]. Interestingly in the studies included in the present review PEA and asystole were the most frequently reported initial CA rhythms [4,8]. This may because most SAH induced arrest is due the sudden rise of ICP resulting in respiratory arrest followed by hypoxia and asystole. It is also possible that some patients found in asystole may have initially have had VF. Several patients with an initial rhythm of VF were in fact included in the study by Toussaint and colleagues [3]. It is probable that there is underreporting in the literature of successfully resuscitated SAH patients with VF as the initial rhythm.

Despite a high likelihood of ROSC few patients survive to be discharged from the hospital [4]. Survival of SAH patients seems to be related to the duration of the cardiac arrest, which is not surprising since the latency to ROSC is an important predictor of outcome in CA patients overall [15]. Few patients requiring prolonged resuscitation i.e. latency to ROSC longer than 20 minutes survive. Indeed most reported survivors have had their arrest in the hospital or in the ambulance. The outcome of patients with SAH who experience a cardiac arrest in the hospital is reported by Toussaint and colleagues and recently by Walmuller and colleagues [3,12]. As previously discussed underreporting of outcomes of SAH patients experiencing cardiac arrest in the hospital is likely.

In most studies SAH patients that experienced a cardiac arrest had more a severe type of SAH with large bleeds and intraventricular clots. These patients were admitted deeply comatose with World Federation of Neurosurgical Surgeon (WFNS) grades of 4–5, a patient group that even without a secondary hit in form of a cardiac arrest with additional ishaemia, have a very high mortality [21]. In one study 13% of SAH patients presenting with a WFNS grade of V had a favourable outcome at 6 months [21]. It has been suggested, however, that the outcome of patients with poor grade SAH has improved during recent years [22].

In studies included in this review neurosurgical care was mainly directed to patients with short delays to ROSC, likely because of poor clinical condition and lack of brain stem reflexes in those with a prolonged cardiac arrest [8]. An important aspect is the possibility of organ donation in SAH patients who are declared brain dead or, in some countries as a part of non-heart beating organ donation programs [23].

Very little can be said about the effect of ALS measures in patients with CA due to SAH. The use of adrenaline seems to be common. Adrenaline may cause hypertension after ROSC and this might even be harmful due to the high risk of rebleed in SAH patients. Firm conclusions abut these issues are difficult to make and very challenging if not impossible to study given the overall rarity of SAH causing CA and because diagnosing SAH in the pre-hospital setting.

The results of this review support the vigilant use of CT scanning in patients following OHCA [24]. Especially, since headache is a common symptom prior to the arrest but it is not 100% sensitive, as shown in the study by Inamasu and colleagues where 50% of the OHCA patients with SAH did not have symptoms prior to the arrest [13].

### Study limitations

The present review has some limitations. Firstly, few of the included studies had been conducted according to the Utstein Guidelines hindering the comparison of results. Secondly, most studies originated from Japan and it may be debated how well these results are applicable to centres in other parts of the world. Thirdly, this review included 3 papers by Inamasu and colleagues originating from the same hospital and from partly overlapping time periods. It is therefore possible that the patient material is partly the same leading overrepresentation of their findings.

## Conclusion

Cardiac arrest is a fairly common complication following severe SAH and these patients are encountered both in the pre-hospital and in-hospital setting with variable frequency. Survival is possible if the arrest occurs in the hospital and the latency to ROSC is short. In OHCA outcome seems to be uniformly poor despite initially successful resuscitation.

## Competing interests

The authors declare that they have no competing interests.

## Authors' contributions

MBS and MP designed the study and MBS performed the literature search. MBS an MP individually reviewed the included studies. MBS drafted the manuscript and it was revised by MP. Both authors read and approved the final manuscript.
